# Semen Quality in a Large Cohort of Males Living in Highly Polluted Areas of Campania Region in Southern Italy with a Focus on the Role of Cadmium Exposure

**DOI:** 10.3390/jcm15134949

**Published:** 2026-06-25

**Authors:** Cristina de Angelis, Francesco Garifalos, Davide Menafra, Paolo Chiodini, Giacomo Galdiero, Mariangela Piscopo, Tonia Romano, Nunzia Verde, Antonella Giarra, Marco Trifuoggi, Erminio Massimo Crescenzo, Chiara Simeoli, Mariarosaria Negri, Claudia Pivonello, Annamaria Colao, Rosario Pivonello

**Affiliations:** 1 Dipartimento di Medicina Clinica e Chirurgia, Sezione di Endocrinologia, Diabetologia ed Andrologia, Unità di Andrologia e Medicina della Riproduzione, Sessualità e Affermazione di Genere, Università Federico II di Napoli, 80131 Naples, Italy; cristinadeangelis83@hotmail.it (C.d.A.); francesco.garifalos@gmail.com (F.G.); menafradavide@gmail.com (D.M.); galdiero.giacomo@hotmail.it (G.G.); mariangelapiscopo@hotmail.com (M.P.); toniaroma97@gmail.com (T.R.); dottverdenunzia@gmail.com (N.V.); 2 Medical Statistics Unit, University of Campania “Luigi Vanvitelli”, 80138 Naples, Italy; paolo.chiodini@unicampania.it; 3 Dipartimento di Scienze Chimiche, Università Federico II di Napoli, 80126 Naples, Italy; antonella.giarra@unina.it (A.G.); marco.trifuoggi@unina.it (M.T.); 4 Dipartimento di Medicina Clinica e Chirurgia, Sezione di Endocrinologia, Diabetologia ed Andrologia, Università Federico II di Napoli, 80131 Naples, Italy; erminiomassimocrescenzo@gmail.com (E.M.C.); simeolichiara@gmail.com (C.S.); negrimariarosaria@yahoo.it (M.N.); colao@unina.it (A.C.); 5 Dipartimento di Psicologia e Scienze della Salute, Università Telematica Pegaso, 80132 Naples, Italy; 6 Department of Public Health, Federico II University, 80131 Naples, Italy; claudia.pivonello@unina.it; 7 Unesco Chair for Health Education and Sustainable Development, Federico II University, 80131 Naples, Italy

**Keywords:** environment, cadmium, endocrine disruptors, male reproduction, fertility, semen quality, Land of Fires, Campania, Italy

## Abstract

**Background/Objectives:** The “Land of Fires” (LF) in the Campania Region has attracted considerable attention due to massive environmental contamination deriving from decades of illegal disposal, burial, and burning of urban, industrial, and toxic waste. Cadmium (Cd) has been repeatedly proven to affect male reproductive function by a plethora of endocrine and non-endocrine mechanisms. The scientific literature is almost devoid of large studies addressing semen quality in this area, particularly by directly correlating seminal parameters to objectively measured pollutant burden in biological samples. Therefore, the aim of the current study was to comprehensively evaluate semen quality of males of reproductive age living in the LF, by correlating seminal parameters to cumulative local male reproductive tract Cd burden objectively quantified in whole semen samples. **Methods**: The current single-center, observational, cross-sectional study evaluated semen quality in 493 males aged 14–50 (29.07 ± 7.17) years living in three LF municipalities. Moreover, the association of semen quality with whole semen Cd (sCd) levels measured by inductively coupled plasma mass spectrometry (ICP-MS) was addressed in a subgroup of participants; semen samples suitable for semen Cd measurements were available from 383/493 (77.7%) participants of the total cohort, and all analyses involving semen Cd were performed within the measured subset. **Results**: In the total cohort, seminal parameters were as follows: semen pH 8.32 ± 0.3, semen volume 3.13 ± 1.67 mL, sperm concentration 37.58 ± 30.18 × 10^6^/mL, total count 111.2 ± 104 × 10^6^/ejaculate, total motility 56.83 ± 16.09%, progressive motility 50.22 ± 16.63%, in situ motility 6.72 ± 3.43%, immotile spermatozoa 43.07 ± 15.88%, normal morphology 7.97 ± 4.02%, and viability 64.75 ± 15.34%. Prevalence of normozoospermia and pathological seminal parameters was as follows: normozoospermia 66.5% (328/493), pathological seminal parameters 33.5% (165/493), specifically, oligozoospermia 14% (69/493), cryptozoospermia 0.8% (4/493), azoospermia 2.2% (11/493), asthenozoospermia 3% (15/493), teratozoospermia 0.6% (3/493), oligo-astheno-teratozoospermia 6.1% (30/493), necrozoospermia 5.7% (28/493), and different combined seminal parameters alterations 7.1% (35/493). Whole semen Cd was below (undetectable) or above (detectable) the limit of detection (LoD) (0.2 μg/L) in 66.6% (255/383) and 33.4% (128/383) whole semen samples, respectively. In samples with detectable sCd, sCd level was below or above the median value (0.76 μg/L; min–max 0.1–5.95 μg/L) in 23.4% (30/128) and 76.6% (98/128) whole semen samples, respectively. Participants with detectable sCd levels had a significantly reduced sperm total count (93.28 ± 84.88 × 10^6^/ejaculate vs. 113.2 ± 101.5 × 10^6^/ejaculate; *p* = 0.037), and normal morphology (7.29 ± 3.71% vs. 8.23 ± 3.91%; *p* = 0.034), and a significantly lower prevalence of normozoospermia (60.2% vs. 72.2%; *p* = 0.02) and significantly higher prevalence of pathological seminal parameters (39.8% vs. 27.8%; *p* = 0.02), specifically, a significantly higher prevalence of oligozoospermia (21.1% vs. 12.6%; *p* = 0.036) than those with undetectable sCd levels. Whole semen Cd levels were significantly higher in participants with pathological seminal parameters (1.08 ± 0.84 μg/L vs. 0.93 ± 0.74 μg/L; *p* = 0.037) than those with normozoospermia. Participants with sCd levels above the median value (N = 98) had a significantly reduced sperm concentration (29.12 ± 24.84 × 10^6^/mL vs. 43.62 ± 29.55 × 10^6^/mL; *p* = 0.015) and displayed a trend towards reduced sperm normal morphology (6.92 ± 3.38% vs. 8.55 ± 4.49%; *p* = 0.057) than those with sCd levels below the median value (N = 30). Moreover, participants with sCd levels above the median value (N = 98) had a significantly reduced sperm concentration (29.12 ± 24.84 × 10^6^/mL vs. 35.3 ± 26.29 × 10^6^/mL; *p* = 0.03), total count (85.77 ± 80.52 × 10^6^/ejaculate vs. 113.2 ± 101.5 × 10^6^/ejaculate; *p* = 0.008) and normal morphology (6.92 ± 3.38% vs. 8.23 ± 3.91%; *p* = 0.006), and a significantly lower prevalence of normozoospermia (57.1% vs. 72.2%; *p* = 0.008) and significantly higher prevalence of pathological seminal (42.9% vs. 27.8%; *p* = 0.008), specifically, a significantly higher prevalence of oligozoospermia (23.5% vs. 12.6%; *p* = 0.014) than those with undetectable sCd levels. **Conclusions**: The results of the current study demonstrate an association between the environmental Cd exposure and the impairment of seminal parameters, with a significantly poorer semen quality in participants with detectable sCd, and, more markedly, in those with sCd level above the median value, compared to participants with undetectable sCd, although subgroups comparisons highlighted a homogeneous profile in major confounders including age, BMI, and smoking habits among subgroups of participants with different sCd burden.

## 1. Introduction

The worldwide human fertility rate has progressively declined over the last six decades [1]. According to demographic projections, in 2050 the rate will drop below the population replacement threshold of 2.1 children per woman worldwide [2]. In Italy, the fertility rate will stand at 1.36 in 2050 and drop down to 1.5 in 2100 [2]. The Increase in the economic burden of parenthood and socio-cultural cues are unlikely the unique factors accounting for such a dramatic trend, suggesting that additional factors might affect global reproductive health; this scenario raises an important public health concern, prompting investigations into the possible underlying determinants of reproductive dysfunction, with particular alarm on the potential impact of exposure to toxic pollutants [3].

Within the estimated 48.5 million infertile couples worldwide [4], male infertility has been reported, on average, to be solely responsible for 20–30% and contributes to about 50% of infertility cases, with the distribution of infertility due to the male factor ranging from 20% to 70% in different countries [5]. Since the ’90s, parallel to declining fertility rates, a significant overall deterioration of semen quality has been reported in several populations worldwide, particularly related to sperm concentration and total sperm count in industrialized Western countries [6]. Although the causes underlying declining male fertility potential are likely multifactorial, in line with correlative epidemiological data in humans, exposure to environmental pollutants is claimed to significantly contribute, despite the fact that a causal relationship is mainly substantiated by experimental in vitro and in vivo animal models [7,8,9].

A direct consequence of global industrialization has been the exponential rise in environmental pollutants, particularly in countries with poorer surveillance and regulations. The area known as “Land of Fires” (LF), comprising ninety municipalities located in the Province of Naples and Caserta within the third most populous and the second most densely populated Italian Region, the Campania Region, in Southern Italy, has attracted considerable scientific and media attention because of widespread environmental contamination with compounds displaying endocrine-disrupting properties, such as dioxins, polychlorinated biphenyls, hydrocarbons, and heavy metals. The impressive local high environmental impact (HI) stemming from decades of illegal disposal, burial, and uncontrolled burning of urban, industrial, and toxic waste in this area, raised pervasive concerns about the potential health consequences of such contamination [10].

Among the compounds contaminating the LF environment, heavy metals are classified as systemic toxicants and represent a considerable public health threat because of their persistence, bioaccumulation, and biomagnification through the food chain [11]. Despite a few studies attempting to address the general health status of residents within the LF, relatively scarce investigation has been performed so far concerning semen quality and male reproductive function. In particular, a geochemical ecological study performed in the metropolitan area of Naples described a strong association between high concentrations of heavy metals in soils and poor seminal parameters [12]. Moreover, two biomonitoring studies performed on small cohorts of men living in HI areas of the Campania Region reported reduced sperm motility and increased seminal oxidative stress markers, spermatozoa DNA fragmentation [13], and telomere length [14] compared to residents of low environmental impact (LI) areas.

Cadmium (Cd), a soft silver-white metal, is a ubiquitous environmental pollutant of particular concern, mainly derived from anthropogenic activities such as industrial processes, fossil combustion, and waste incineration [15,16]. In non-occupationally exposed populations, the main Cd sources are cigarette smoke and contaminated water and food (particularly cereals, grains, leafy vegetables, potatoes, and offal) [15,16]. Due to bioaccumulation in organic matter, very long biological half-life, and poor clearance in the human body [15,16], Cd burden progressively gathers in the liver and kidney, as well as in male reproductive organs, comprising testis and epididymis, and consequently in semen [17,18], boosting worries for the effects of Cd on semen quality and male fertility, even at low-level environmental chronic exposures [19]. Indeed, several lines of evidence in experimental models clearly documented that Cd exposure might jeopardize testicular structure and function by compromising both steroidogenesis and spermatogenesis through a repertoire of endocrine and non-endocrine mechanisms that include damage to testicular vascular endothelium and the blood–testis barrier, inflammation and apoptosis, Sertoli and Leydig cell-targeted cytotoxicity, oxidative stress in Leydig and germ cells, DNA fragmentation, epigenetic changes, and hypothalamus–pituitary–testis axis disturbances [20]. Clinical studies provide more heterogeneous findings, due to shortcomings related to different study design (mostly retrospective) and methodological settings, small sample size, and frequent lack of adjustment for potential confounders. Nevertheless, the majority of studies highlight an association of environmental Cd exposure with poor semen quality, male infertility, and adverse reproductive outcomes. However, the male general population, unselected for fertility status and from now onwards referred to as “general population” in the current study, is frequently disregarded as a target [8,20,21].

To the best of the authors’ knowledge, few studies evaluated semen quality in men living in HI areas within the Campania Region, and no large-scale confirmatory case series are available that specifically address the potential impact of environmental Cd exposure, objectively measured in biological matrices, on semen quality. Therefore, the aim of the current single-center, observational, cross-sectional study was to comprehensively evaluate semen quality in males of reproductive age living in three municipalities (Acerra, Afragola, and Giugliano in Campania) belonging to the LF, by correlating seminal parameters to cumulative local male reproductive tract Cd burden objectively quantified in whole semen samples, for the first time in a large homogeneous cohort with assessment of potential confounders.

## 2. Materials and Methods

### 2.1. Study Design

The current research study is a single-center, observational, cross-sectional study. A preliminary descriptive analysis of anthropometric, lifestyle-related and seminal parameters was performed, and prevalence of pathological seminal parameters, namely, parameters below the reference cut-off value according to the guidelines of the 2010 World Health Organization (WHO) Laboratory Manual for the Examination and Processing of Human Semen [22], was determined in the total cohort. Whole semen Cd (sCd) levels were correlated with anthropometric, lifestyle-related, and seminal parameters. In a first subanalysis of data, sCd level was set as dichotomous variable, and participants of the total cohort with available Cd measurement were grouped as being below (undetectable) or above (detectable)the sCd limit of detection (LoD), in order to detect differences in continuous variables and prevalence of pathological seminal parameters; in a second subanalysis, participants were grouped as being below or above the median sCd level or having undetectable Cd levels, to further characterize the relationship between semen quality and Cd burden. Lastly, exclusively in the subgroup of participants with detectable sCd, a comparative analysis was performed in smokers and non-smokers, to address the impact of smoking status on semen quality and, particularly, on sCd burden.

### 2.2. Participants Recruitment

Participant recruitment and the research study were performed in line with the principles of the Declaration of Helsinki, and upon approval by the Ethical Committee of the “Federico II” University of Naples (Prot. N°158/19). The study cohort included Caucasian males of reproductive age residing in the Campania Region, enrolled within a two-year (2017–2019) research project entitled “Exposoma e Plurifocalità nella Prevenzione Oncologica”. An awareness campaign for infertility and testis cancer prevention was promoted by the Department of Clinical Medicine and Surgery, Section of Endocrinology, Diabetology, Andrology and Nutrition, Unit of Andrology and Medicine of Reproduction, Sexuality and Gender Affirmation – Federico II University of Naples, in HI areas within the Campania Region, identified by Campania Region Environmental Protection Agency (ARPAC) reports as being characterized by the highest density of illegal toxic waste dumping sites, and marked by frequent, uncontrolled waste incineration. The awareness campaign was disseminated through publication on the recruiting center’s official website, by sharing informative material through social media platforms and by locally distributed flyers; additionally, participants were sequentially recruited by direct telephone contact using telephone directories, and with the support of general practitioners and community pharmacists. The totality of participants was enrolled on a voluntary basis and received no financial remuneration, compensation, or other incentives for their participation.

### 2.3. Inclusion and Exclusion Criteria

The inclusion criterion was residence for at least 10 years in municipalities belonging to HI areas of the Campania Region; specifically, the research study HI area of interest comprised three municipalities (Acerra, Afragola, and Giugliano in Campania) located in the Province of Naples and Caserta, belonging to the LF. The years of residence and the daily permanence within the study area were recorded to provide an indirect estimate of cumulative environmental exposure. The exclusion criteria included the following: (1) psychological and/or psychiatric disorders preventing from understanding the nature of the study or from providing informed con-sent for participation to the study; (2) chronic endocrine or systemic diseases potentially affecting semen quality and male fertility; (3) use of medications or pathological conditions with known effects on seminal parameters potentially affecting semen analysis; (4) history of or concurrent non-therapeutic use of psychotropic substances and/or recreational drugs; (5) history of or concurrent use of performance-enhancing agents or antiandrogen medications; (6) history of or concurrent alcoholism, or suspicion of alcohol abuse. No “a priori” selection based on the presence or absence of infertility and/or andrological disorders was applied as a criterion for participant enrolment; the functional status of the hypothalamus–pituitary–gonadal axis was not considered in the current study. Inclusion of adolescents was intended to capture early-life environmental exposure effects on reproductive health, given the increased susceptibility during developmental stages; moreover, the awareness campaign within which the current study was designed had the specific aim to provide evidence on the importance of early prevention for infertility and testis cancer, the most frequent solid malignancy in very young males. A written informed consent for participation in the study was obtained upon enrolment of participants attending the recruiting center; a written informed consent was obtained from a legal representative in case of underage participants. Upon enrolment, a unique sequential code was allocated to each participant by the enrolling clinician for anonymity; the examining biologist performed semen analysis blinded to participant identity, residential municipality, and clinical characteristics.

### 2.4. Clinical Procedures and Data Collection

Accurate clinical and pharmacological anamnesis and complete physical and andrological examination, comprising evaluation of testicular volume by Prader orchidometer and testis ultrasound, were performed. Anthropometric parameters: weight, height, and body mass index (BMI) were recorded. The height was measured to the nearest 1 mm by using a stadiometer (Seca, Hamburg, Germany) and the weight was measured to the nearest 0.1 kg by using an electronic scale. The BMI was calculated as weight (kg) divided by the square of height (m). Specifically trained personnel administered structured questionnaires face-to-face to collect data concerning lifestyle-related parameters, particularly smoking habits and occupational history with self-reported occupational exposure to toxic chemicals. Non-smokers were defined as participants who had smoked less than 100 cigarettes in their lifetime (never-smokers) or participants who had quit smoking more than 2 years before the interview; current smokers were defined as participants who had smoked at least 100 cigarettes in their lifetime and who still smoked cigarettes or had quit smoking ≤2 years before the interview. Smoking habits were quantitatively evaluated based on self-reported information by using the cigarette pack/year parameter (1 pack/year = 365.24 packs of cigarettes or 7305 cigarettes; number of pack/years = packs smoked per day × years as a smoker) [23].

### 2.5. Semen Analysis

Semen samples were collected on-site at the recruiting center by masturbation directly into a sterile wide-mouth metal-free container after 3–5 days of sexual abstinence. Use of medications or pathological conditions with known effects on seminal parameters were accounted for at the time of semen collection by anamnestic interview; whenever possible, semen analysis was deferred, otherwise the participant was excluded from the study. Abstinence period and spillage, if any, were recorded. Semen quality was analyzed according to WHO 2010 guidelines [22]. After collection, ejaculates were incubated at 37 °C and allowed to liquefy before processing; semen analysis was conducted within 60′ from semen collection. The following seminal parameters were included in the analysis: semen pH, semen volume (mL), sperm concentration (n × 10^6^/mL), sperm total count (n × 10^6^/ejaculate), sperm total motility (%), sperm progressive motility (%), sperm in situ motility (%), immotile spermatozoa (%), sperm normal morphology (%), and sperm viability (%). Sperm count and motility were evaluated with the Makler Counting Chamber (Sefi Medical Instruments, Haifa, Israel) using a Nikon E200 optical microscope (Nikon, Tokyo, Japan) (20× magnification, phase-contrast); sperm morphology was evaluated after Giemsa staining (100× magnification, bright field); sperm viability was evaluated after Eosin–nigrosine staining (100× magnification, bright field). The WHO 2010 criteria for normozoospermia were as follows: sperm total count ≥ 39 × 10^6^/ejaculate or sperm concentration ≥15 × 10^6^/mL; sperm progressive motility ≥ 32% or sperm total motility ≥ 40%; sperm normal morphology ≥ 4%; sperm viability ≥ 58% [22]. The totality of semen samples was analyzed in the same laboratory by the same trained and experienced operator. The laboratory and the operators take part in an international external quality control program for semen analysis on a quarterly basis, provided by UK Neqas International Quality Expertise. After semen analysis, aliquots of 500 µL of whole semen were collected and stored in metal-free tubes at −80 °C for subsequent Cd quantitative determination.

### 2.6. Cadmium Analysis

Whole semen samples suitable for sCd measurements were not available for all participants; therefore, quantitative determination of Cd by inductively coupled plasma mass spectrometry (ICP-MS) (Aurora M90 instrument) (Bruker, Billerica, MA, USA) was performed in whole semen samples from 383/493 participants of the total cohort. ICP-MS was targeted specifically to Cd detection. Briefly, 500 µL of whole semen sample were transferred into glass tubes, and 1 mL of ultrapure nitric acid (HNO_3_ ≥ 69%, *v*/*v*, TraceSELECT^®^)(Solstice Advanced Materials, Morris Plains, NJ, USA) was added. Sealed tubes were subjected to acid oxidative digestion using an automated microwave system (DISCOVER SP-D)(CEM, Matthews, North Carolina, USA) by the following protocol: 3′ ramp time from room temperature (RT) to 160 °C; 2′ hold time at constant 160 °C temperature; and 2′ cooling time from 160 °C to 80 °C, and from 80 °C to RT with no auxiliary cooling control. Once at RT, HNO_3_ 2% (*v*/*v*) was added to the mix, brought to the final volume of 10 mL. A calibration curve for Cd was obtained from certified standard solutions, and sample Cd concentration was determined by comparison; final Cd concentrations were reported as μg/L. The LoD and limit of quantification were calculated as the blank signal plus three or ten times its standard deviation, respectively. LoD for Cd was 0.2 μg/L. Recovery was calculated to be within the range of 70–120%. The rationale for using sCd is supported by multiple previous studies from the literature demonstrating that Cd preferentially accumulates in the testis, and that its concentration in the male reproductive tract is not correlated to blood or urinary concentrations [20,24,25], therefore indicating sCd as the most biologically relevant marker of local exposure and the most meaningful biomarker for the assessment of Cd impact on reproductive outcomes.

### 2.7. Statistical Analysis

Statistical analysis was performed using GraphPad Prism version 10 (Dotmatics, Boston, MA, USA) and IBM SPSS version 29 (IBM Corp., Armonk, NY, USA) Statistics softwares. The sample size was determined pragmatically according to the number of eligible participants recruited during the project period. Missing data were present for lifestyle variables (N = 461/493) and for sCd measurements (N = 383/493). Missing data were handled using a complete-case approach, without imputation, and the available sample size is reported for each analysis. Distribution of continuous variables was assessed using the D’Agostino–Pearson normality test. Descriptive statistics are reported as mean ± standard deviation (SD) for normally distributed variables and as median (min-to-max) for non-normally distributed variables. Categorical variables are presented as absolute numbers and percentages. Pearson or Spearman correlation test was applied to determine the linear relationship between two continuous variables or between ranked values of categorical variables, respectively. Between-group comparisons of continuous variables were performed using the unpaired Student’s *t*-test or ANOVA for normally distributed data, and the Mann–Whitney or Kruskal–Wallis test for non-normally distributed data. Comparisons of categorical variables were performed using the Chi-squared test or Fisher’s exact test, as appropriate. Statistical analyses are unadjusted. For the main semen-related between-group comparisons, unadjusted mean differences with 95% confidence intervals were calculated for continuous variables, and unadjusted risk differences with 95% confidence intervals were calculated for categorical semen-quality outcomes. In analyses involving three sCd groups, participants with undetectable sCd were used as the reference category. Potential confounding by age, BMI, smoking habits, duration of residence or mean time spent/day in HI areas, and self-reported occupational exposure, selected a priori according to their biologically plausible impact on semen quality and to the fact that they are associated and/or have been reported to influence Cd burden [20,26], was explored through subgroup comparisons and correlation analyses. All statistical tests were two-sided, and statistical significance was set at *p* < 0.05.

## 3. Results

### 3.1. Total Cohort Anthropometric, Lifestyle-Related, and Seminal Parameters

Four hundred ninety-three males aged 14–50 (29.07 ± 7.17) years with a BMI of 25.82 ± 4.1 Kg/m^2^, a mean duration of residence in HI areas of Campania Region of 25.59 ± 9.13 years, and a mean time spent/day in HI areas of 16.68 ± 5.85 h/24 h were recruited within the current study; the first participant was recruited on 26 August 2019. Among recruited participants, 6.5% (32/493) refused to be interviewed concerning lifestyle-related parameters; therefore, analysis of lifestyle-related parameters was carried out in 461 participants responding to the interview; among these, 52.7% (243/461) were non-smokers and 47.3% (218/461) were current smokers with a mean number of cigarettes smoked/day of 11.02 ± 8.06, accounting for 6.08 ± 8.47 cigarette pack years; occupational exposure to toxic chemicals was self-reported by 11.7% (54/461) participants (Table 1).

Seminal parameters were as follows: semen pH 8.32 ± 0.3, semen volume 3.13 ± 1.67 mL, sperm concentration 37.58 ± 30.18 × 10^6^/mL, total count 111.2 ± 104 × 10^6^/ejaculate, total motility 56.83 ± 16.09%, progressive motility 50.22 ± 16.63%, in situ motility 6.72 ± 3.43%, immotile spermatozoa 43.07 ± 15.88%, normal morphology 7.97 ± 4.02%, and viability 64.75 ± 15.34%. Prevalence of normozoospermia and pathological seminal parameters was as follows: normozoospermia 66.5% (328/493), pathological seminal parameters 33.5% (165/493), specifically, oligozoospermia 14% (69/493), cryptozoospermia 0.8% (4/493), azoospermia 2.2% (11/493), asthenozoospermia 3% (15/493), teratozoospermia 0.6% (3/493), oligo-astheno-teratozoospermia 6.1% (30/493), necrozoospermia 5.7% (28/493), and different combined seminal parameters alterations 7.1% (35/493) (Table 2).

### 3.2. Anthropometric, Lifestyle-Related, and Seminal Parameters, According to Whole Semen Cadmium Levels

**Figure 1:**
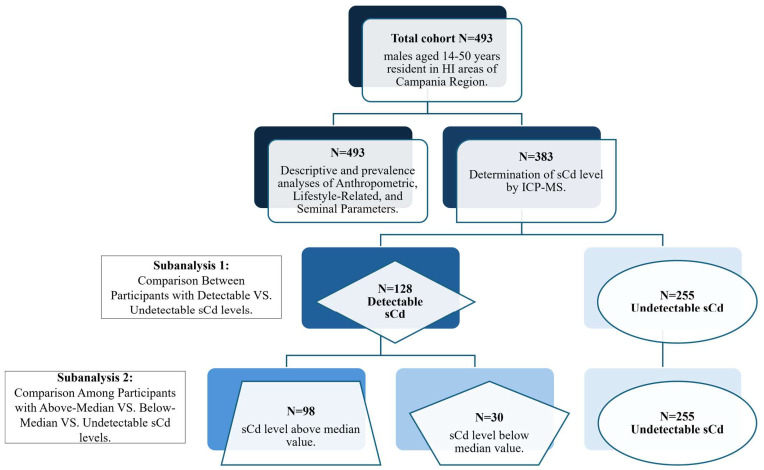
Flow diagram summarizing participant inclusion, availability of whole semen cadmium (sCd) measurements, and schematic representation of statistical analyses and subanalyses performed, relative to continuous variables and prevalences of anthropometric, lifestyle-related, and seminal parameters. Abbreviations: HI, high environmental impact; sCd, whole semen cadmium; ICP-MS, inductively coupled plasma mass spectrometry.

A flow diagram summarizing participant inclusion and the availability of sCd measurements by ICP-MS is provided (Figure 1).

**Figure 2:**
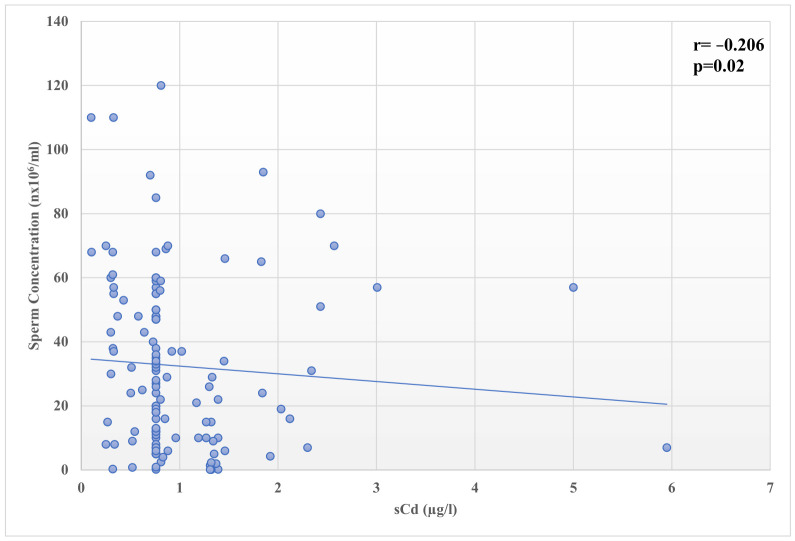
Distribution of whole semen cadmium (sCd) levels plotted against sperm concentration (r = −0.206; *p* = 0.02).

Semen samples suitable for sCd measurements were not available for all participants; Cd level was determined in 77.7% (383/493) whole semen samples of the total cohort. Comparison of participants with available sCd measurements with those without available sCd measurements for key anthropometric, lifestyle-related, and semen parameters is reported in Supplementary Table S1. All analyses involving sCd were performed within the measured subset, with overtly reported sample sizes for each analysis. Whole semen Cd was below (undetectable) or above (detectable) the LoD (0.2 μg/L) in 66.6% (255/383) and 33.4% (128/383) whole semen samples, respectively. In samples with detectable sCd, sCd level was below or above the median value (0.76 μg/L; min–max 0.1–5.95 μg/L) in 23.4% (30/128) and 76.6% (98/128) of the whole semen samples, respectively. In Spearman correlation analysis, the sCd level was negatively correlated with sperm concentration (r = −0.206; *p* = 0.02) (Figure 2); a trend towards a negative correlation was also identified between sCd level and sperm total count (r = −0.171; *p* = 0.054). No correlations were detected between sCd and anthropometric, lifestyle-related, or other seminal parameters.

**Figure 3:**
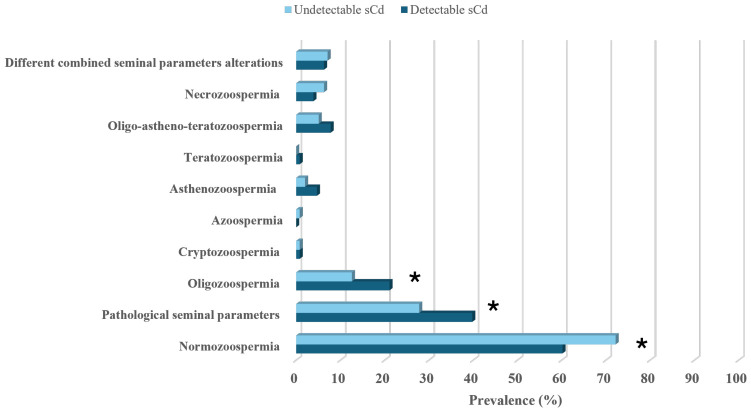
Prevalence of normozoospermia and of pathological seminal parameters in subgroups of participants with detectable (blue bar) and undetectable (light-blue bar) whole semen cadmium (sCd) levels. Values expressed as percentage. Significantly different: normozoospermia * *p* = 0.02; pathological seminal parameters * *p* = 0.02; oligozoospermia * *p* = 0.036.

In the first subanalysis of data, no significant difference was found between participants with undetectable and detectable sCd levels, concerning any of the assessed anthropometric or lifestyle-related parameters. Conversely, participants with detectable sCd levels had a significantly reduced sperm total count (93.28 ± 84.88 × 10^6^/ejaculate vs. 113.2 ± 101.5 × 10^6^/ejaculate; *p* = 0.037) and normal morphology (7.29 ± 3.71% vs. 8.23 ± 3.91%; *p* = 0.034), compared to those with undetectable sCd levels. Consistently, participants with detectable sCd levels had a significantly lower prevalence of normozoospermia [60.2% (77/128) vs. 72.2% (184/255); *p* = 0.02], and a significantly higher prevalence of pathological seminal parameters [39.8% (51/128) vs. 27.8% (71/255); *p* = 0.02], specifically, a significantly higher prevalence of oligozoospermia [21.1% (27/128) vs. 12.6% (32/255); *p* = 0.036] (Table 3, Figure 3). In line with the results of the first subanalysis, participants with pathological seminal parameters had significantly higher sCd levels compared to those with normozoospermia (1.08 ± 0.84 μg/L vs. 0.93 ± 0.74 μg/L; *p* = 0.037).

**Figure 4:**
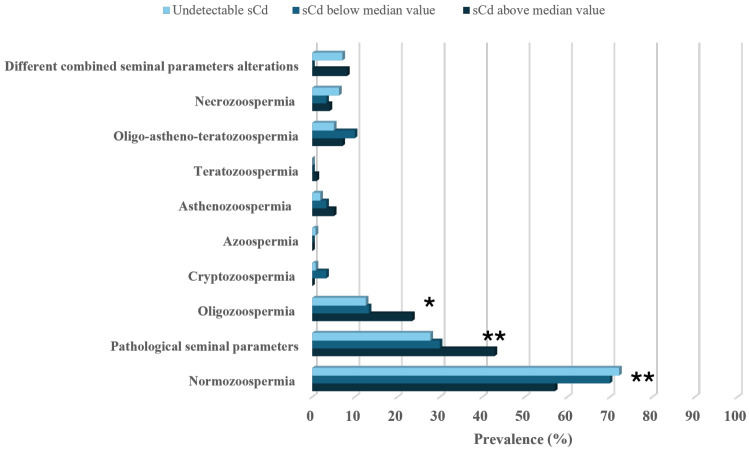
Prevalence of normozoospermia and of pathological seminal parameters in subgroups of participants with whole semen cadmium (sCd) above the median value (dark blue bar), sCd level below the median value (blue bar), and undetectable sCd levels (light-blue bar). Values expressed as percentages. Significantly different in sCd level above the median value vs. undetectable sCd levels: normozoospermia ** *p* = 0.008; pathological seminal parameters ** *p* = 0.008; oligozoospermia * *p* = 0.014.

In a second subanalysis of the data, no significant difference was found among participants with undetectable sCd levels or with sCd levels below or above the median value, concerning any of the assessed anthropometric or lifestyle-related parameters. Conversely, sperm concentration (*p* = 0.017), total count (*p* = 0.024), normal morphology (*p* = 0.015), and prevalence of normozoospermia (*p* = 0.025) of pathological seminal parameters (*p* = 0.025) and, specifically, of oligozoospermia (*p* = 0.037) overall significantly differed among the three groups. In particular, the group-by-group comparison highlighted that participants with sCd levels above the median value had a significantly reduced sperm concentration (29.12 ± 24.84 × 10^6^/mL vs. 43.62 ± 29.55 × 10^6^/mL; *p* = 0.015) and displayed a trend towards reduced sperm normal morphology (6.92 ± 3.38% vs. 8.55 ± 4.49%; *p* = 0.057), compared to participants with sCd levels below the median value. Moreover, participants with sCd levels above the median value had a significantly reduced sperm concentration (29.12 ± 24.84 × 10^6^/mL vs. 35.3 ± 26.29 × 10^6^/mL; *p* = 0.03), total count (85.77 ± 80.52 × 10^6^/ejaculate vs. 113.2 ± 101.5 × 10^6^/ejaculate; *p* = 0.008), and normal morphology (6.92 ± 3.38% vs. 8.23 ± 3.91%; *p* = 0.006), compared to participants with undetectable sCd. Consistently, participants with sCd levels above the median value had a significantly lower prevalence of normozoospermia [57.1% (56/98) vs. 72.2% (184/255); *p* = 0.008] and a significantly higher prevalence of pathological seminal parameters [42.9% (42/98) vs. 27.8% (71/255); *p* = 0.008] and, specifically, of oligozoospermia [23.5% (23/98) vs. 12.6% (27/255); *p* = 0.014], compared to participants with undetectable sCd (Table 4, Figure 4).

Unadjusted effect-size estimates supported the interpretation of the main analyses and are reported in Supplementary Tables S2 and S3. Participants with detectable sCd showed lower sperm total count and normal morphology, and a less favorable distribution of semen-quality categories, compared to participants with undetectable sCd. In the three-group analysis, the largest differences were observed in participants with sCd above the median value versus those with undetectable sCd.

### 3.3. Seminal Parameters and Whole Semen Cadmium Levels, According to Smoking Status

In a subanalysis of data, performed in participants with detectable sCd to address the impact of smoking status on semen quality and, particularly, on sCd burden, no significant difference was found in smokers (N = 45) compared to non-smokers (N = 74), concerning any of the assessed anthropometric, lifestyle-related, or seminal parameter, as well as in the prevalence of normozoospermia or of pathological seminal parameters. sCd levels were also not significantly different in the two subgroups (Table 5).

## 4. Discussion

The findings of the current single-center, observational, cross-sectional study provide compelling evidence supporting an association between environmental Cd exposure, objectively addressed by Cd quantification in a large series of whole semen samples, and semen quality, in males of reproductive age residing for at least 10 years in HI areas of Campania Region, commonly referred to as the LF.

The findings of the current study demonstrated that participants with detectable sCd had poorer semen quality, in terms of significantly reduced sperm total count and normal morphology, and a significantly higher prevalence of pathological seminal parameters and, specifically, of oligozoospermia, compared to participants with undetectable sCd, whereas no significant differences in major confounders including age, BMI, smoking habits, duration of residence or mean time spent/day in HI areas, and self-reported occupational exposure could be identified. Similar findings were detected in participants with sCd levels above compared to below the median value and, more markedly, in those with sCd levels above the median value compared to those with undetectable sCd levels. Consistently, sCd levels were significantly higher in participants with pathological seminal parameters compared to those with normozoospermia.

These results are consistent with a growing body of epidemiological evidence demonstrating an inverse relationship between environmental Cd exposure and male reproductive function [8,20]. In particular, a metanalysis of 13 case-control studies on a total of 1.538 sCd samples retrieved from men with different fertility status [21] and case-control studies [27,28,29] reported a significantly higher sCd burden in infertile men, and significantly higher sCd levels were measured in azoospermic and oligozoospermic infertile men, compared to infertile men with normozoospermia [30,31], supporting a potential role of sCd as a biomarker of male infertility and impaired sperm total count. Moreover, in cohorts of infertile men or male partners of infertile couples, sCd level was negatively correlated with sperm concentration [28,31,32], total count [33], motility [28,31,32,33,34], and normal morphology [31,33,35], whereas little evidence was provided of an association between higher sCd level and an increased prevalence of below-reference sperm viability [36]. Conversely, only a few studies failed to identify any correlation between sCd burden and seminal parameters, probably due to small-sized study populations and/or lack of control for potential confounding variables [37,38].

Studies specifically focusing, as the current investigation, on males from the general population, are very scant and are to some extent heterogeneous in reported results but generally support the assumption that even microdoses of sCd may have an effect on semen quality, by reporting data partially in line with the results presented in the current study. In particular, previous studies highlighted a significant negative correlation of both urinary Cd [39] and sCd [40] with sperm viability, although no correlations with different seminal parameters were detected. In disagreement, a different study including a group of 35 men with unknown fertility status failed to demonstrate any correlation between sCd and seminal parameters [32]. Several factors may account for such discrepancies. First, the lower or extremely low sample sizes [32,39,40] and/or the presence of normozoospermia in the majority of men within the study cohort [32], as compared to the current study, might have led to underestimation of potential significant associations. Second, the different analytical methods employed for Cd detection [32,39,40] might have biased or reduced the precision of participants/Cd level classification within study cohorts; indeed, the current study measured sCd by ICP-MS, which displays a lower detection limit and higher precision [41,42], allowing for a better analytical classification of exposure, and reducing the risk of misclassifying participants as non-exposed, a relevant requisite particularly at lower levels of environmental Cd exposure. Lastly, previous studies comprised populations with a much lower level of environmental Cd exposure (sCd median value 0.092 μg/L; min–max 0.039–0.163 μg/L) [32] and (sCd mean value 0.5 ± 0.3 μg/L; max 1.5 μg/L) [39], as compared to the current study (sCd median value 0.76 μg/L; min–max 0.1–5.95 μg/L). These considerations align well with the results of different studies with comparable levels of Cd exposure with respect to the current investigation and providing similar results [43,44].

In line with the hypothesis of a cumulative effect of Cd exposure on semen quality, the current study also demonstrated a significant negative correlation between sCd levels, as a continuous variable, and sperm concentration, and higher sCd levels were associated with reduced sperm total count and normal morphology, suggesting a potential dose-dependent detrimental effect of Cd on spermatogenesis. This hypothesis is consistent with the results of a different study on men from the general population with a similar sCd burden (sCd mean 0.77 μg/L; min–max 0.48–1.22 μg/L) as compared to the current study, also reporting a negative correlation with sperm concentration and total count [44]. Moreover, as in the current study, higher sCd levels were detected in participants with pathological seminal parameters compared to those with normozoospermia by another Italian population-based study on environmental exposure [43].

Taken together, these data might suggest that Cd exposure not only might exert a dose–response effect on sperm numbers, but it might also be speculated that different patterns of exposure intensity might potentially affect different sperm functions, with only sperm viability being affected by lower levels of exposure and sperm production and morphology by higher levels of exposure. These speculations fit well with experimental findings from animal models. [20]. Indeed, acute Cd chloride (CdCl_2_) intraperitoneal administration to adult rats at a dose of 0.2, 0.4, or 0.8 mg/kg body weight drastically affected daily spermatozoa production only at the highest tested dose [45], and a treatment with a dose of 1 mg/kg body weight resulted in a time-dependent failure of spermiation into the seminiferous tubules [46]. Moreover, treatment of adult rats with a dose of 5 mg/kg body weight of CdCl_2_ significantly reduced sperm concentration, normal morphology and viability, as well as testis and epididymis weight, when administered by oral gavage every other day for 30 days [47], and resulted in permanent sterility when administered as a single bolus by subcutaneous injection [48]. Scarce complimentary observational studies in humans are available, concerning the potential association between Cd exposure and testicular morphostructural alterations, predictive of infertility; however, consistent with experimental CdCl_2_-induced testicular damage, the authors of the current study recently demonstrated, in a different study performed on the same cohort of males, a significant association between sCd burden and testicular morphostructural alterations, including reduced testicular volume and increased prevalence of testicular hypotrophy and varicocele, therefore providing a further bio-mechanistic insight supporting the association of Cd exposure with male reproductive dysfunction [49].

Importantly, whereas in animals the effects of adulthood exposure have been proven to be partly reversible, with seminal parameters and fertility status being rescued upon exposure discontinuation, in particular at low doses, early-in-life Cd exposures have been outlined to exert long-lasting and potentially irreversible reproductive damage [20]. Therefore, time-dependent aspects of Cd exposure, including both the onset timing and the duration of exposure, might also be called into question when considering Cd impact on male reproductive function. Indeed, established experimental evidence in animal models identified a remarkably susceptible window of exposure, even at low chronic doses, during prenatal life up to prepubertal stages of development, likely due to the cytotoxic action targeting proliferating and differentiating Sertoli cells, which are fundamental in orchestrating testicular development and growth, by shaping its future functional competence and spermatogenetic potential [50,51], and due to depletion of germ cells [52]. In the current study, residence of at least 10 years in HI areas of the Campania Region was an inclusion criterion; nevertheless, a very high proportion of participants (371/493; 75.3%) reported to be a resident in the specific HI municipalities from birth, therefore, raising major concerns on the long-term effects of potential in utero exposure to environmental Cd on semen quality and male fertility outcomes in the target population. However, no significant difference was detected in the mean duration of residence, nor in the prevalence of residence since birth among the different sCd burden groups, suggesting that cumulative sCd concentration might be a multifactorial event, and/or that adult exposure to additional sources other than environmental Cd contamination, and/or that further factors of susceptibility might account for the differential local male reproductive tract Cd accumulation within studied participants.

Notably, no significant differences were observed between sCd burden groups with respect to anthropometric or lifestyle-related factors, including smoking habits and self-reported occupational exposure. Although multiple regression analysis was not performed in the current study, this finding may support the hypothesis of a potential direct association of the environmental Cd exposure with the impairment of semen quality. Indeed, tobacco leaves are known to accumulate substantial amounts of Cd, thereby rendering tobacco smoke a primary source of Cd exposure in non-occupationally exposed smokers [15]. It has been estimated that each cigarette delivers approximately 1.7 µg of Cd, of which around 10% is inhaled; consequently, individuals who smoke one pack of cigarettes/day absorb about 1–3 µg of Cd, resulting in an approximately twofold higher Cd burden, as compared to non-smokers [15,16]. Moreover, tobacco smoking has been proven to negatively impact seminal parameters, including sperm concentration, total count, motility, and morphology [26,53], and to elevate sCd concentrations [54], therefore suggesting that tobacco-driven sCd might mediate, at least in part, smoke reproductive toxicity. Nevertheless, in the subanalysis specifically performed to deal with the potential independent effect of tobacco smoking on seminal parameters and to better define the potential source of Cd, by analyzing only participants with detectable sCd levels, no significant differences were found in seminal parameters and sCd burden between smokers and non-smokers; this finding potentially suggests that, in the peculiar studied cohort, smoking would be a less relevant source of Cd, as compared to the potential environmental source. Nevertheless, given the small size of the subgroups in the relative subanalysis and the possibility of limited statistical power, this hypothesis should be considered cautiously. Moreover, Cd may also be absorbed from alcoholic beverages; however, alcohol consumption constitutes a significant source of metal exposure only in heavy drinkers [55]; in the current study, reported history of or concurrent alcoholism, or suspicion of alcohol abuse, was set as an exclusion criterion, therefore limiting the impact of alcohol intake as a source of Cd in the current cohort.

Research on experimental models steadily deciphered the multiple pathways of Cd reproductive toxicity: impacting testicular morphostructure, endocrine function, and spermatogenesis, comprising both endocrine and non-endocrine mechanisms such as testicular vascular endothelium and blood–testis barrier functional and structural damage, structural damage to the seminiferous epithelium, inflammation and apoptosis, Sertoli and Leydig cells-targeted cytotoxicity, oxidative stress in Leydig and germ cells, mediated by mimicry and interference with essential ions, genetic instability induced by DNA fragmentation and inhibition of DNA damage repair systems, epigenetic changes, and hypothalamus–pituitary–testis axis derangement [20,56]. Among these, a central and intriguing mechanism is represented by Cd interference with two tightly interconnected systems, namely, redox status and zinc (Zn) and selenium (Se) action and metabolism [20]. Due to a similar chemical structure to Zn, Cd ions get preferential access and accumulate in specific anatomical districts by exploiting Zn transporters, whose expression is notably enriched in the male genital tract, including the testis [57].I Indeed, increased Zn transporter expression has been associated with increased testicular Cd uptake and sensitivity to Cd-induced testis injury in animal models [58]. Moreover, Cd promotes excessive reactive oxygen species (ROS) formation and oxidative stress by impairing ROS scavenger function, through the displacement of Zn from antioxidant enzymes and directly through binding to sulfhydryl groups of ROS scavengers [59,60]; this twofold action leads to excess protein oxidation, lipid peroxidation, and DNA damage in germ cells and spermatozoa, exacerbating cellular damage and, ultimately, cell death [20]. Lastly, Cd has been proven to inhibit Zn intestinal absorption [61,62] and, at higher levels of exposure, to increase Se urinary excretion [63], therefore being capable of decreasing their systemic concentrations. Both Zn and Se are pivotal for male reproductive function, by fueling a conspicuous number of processes such as testosterone production, spermatogenesis, semen quality, and fertility [57,64,65,66]. A wide body of evidence in animals also highlights that Zn and Se treatment might efficiently reverse and/or protect the testis from Cd poisoning [20]. Although the status of essential metals was not addressed in the current investigation, differential Zn and Se intake through diet may not be excluded as an additional potential contributor to sCd accumulation at the high environmental exposure level of the study cohort, and the maintenance of adequate supplementation might be considered as a strategy to empower protection from Cd reproductive toxicity.

Collectively, the mechanistic experimental evidence supports the pathophysiological reasonableness of the associations reported in the current cohort between sCd burden and impaired sperm total count and morphology, although inference of causality is clearly prevented by the observational and cross-sectional nature of the study. Interestingly, no correlations were detected in the studied cohort with reference to sperm motility, partially in contrast with some previous human studies on infertile men [28,31,32,33,34], but it was consistent with the very few data focused, as the current investigation, on males from the general population [32]. In addition, a 2 h in vitro treatment of human spermatozoa with 0.5 μM CdCl_2_ has been shown to significantly reduce sperm progressive and hyperactivated motility [67,68], likely through a direct interaction with sperm head and neck membranes, with a higher binding signal on the sperm neck, which was resistant to scavenging approaches with either hypo-osmotic swelling or reduced glutathione [67]. This experimental evidence highlights that at least a fraction of sCd content might be directly anchored to the sperm cell surface, and, particularly, that the extent of CdCl_2_ accumulation on the sperm head and neck significantly correlates with sCd levels [67]. The discrepancy between epidemiological studies on males from the general population and in vitro studies on human spermatozoa may reflect differences between chronic environmental exposure and acute experimental conditions or suggest that Cd may primarily affect earlier stages of spermatogenesis rather than post-testicular sperm function; nevertheless, experimental evidence supports such a direct toxic effect on mature spermatozoa, potentially blunting sperm fertilizing capability.

From a clinical standpoint, it is noteworthy that, in the studied cohort of males from the general population within the HI study area, detectable sCd levels were associated with a higher prevalence of pathological semen profile, including oligozoospermia, which was further strengthened by subgroup analyses showing the most pronounced impairment in participants at the highest sCd burden group. Another relevant aspect of the study is the relatively high absolute prevalence of semen abnormalities in the overall cohort (33.5%), which further increased (39.8%) when restricting the analysis to participants with detectable sCd, despite the lack of selection based on fertility status. Although not specifically correlated to fertility status, these findings funnel wider implications; while even gross changes in sperm counts are not necessarily linked to corresponding changes in fertility trends, since successful pregnancy might still occur in case of low sperm counts, such a striking impairment of semen quality in the overall cohort and, particularly, in participants with higher sCd, might result in longer waiting time to pregnancy, and, on a long-term basis, might eventually result in the observed decline in fertility trends [69], supporting the hypothesis that chronic exposure to environmental pollutants, including heavy metals, may contribute to male reproductive dysfunction at the population level.

The current study has several strengths. To the best of the authors’ knowledge: (1) this is among the first large-scale investigations addressing Cd burden directly in whole semen samples using a highly sensitive analytical technique, ICP-MS, and correlating sCd burden with seminal parameters; (2) this is the largest study specifically evaluating semen quality in a relatively homogeneous cohort of males with a long-term residency in well-characterized HI areas of the Campania Region, therefore providing a relevant real-world context for environmental exposure assessment; (3) this is also the largest study recruiting from the general population, which does not limit the generalization of the current findings, although the potential for selection bias due to recruitment strategies (voluntary participation in an awareness campaign disseminated through social media, distribution of flyers, direct phone contact, and support from general practitioners and community pharmacists) should be acknowledged as a potential limitation to the representativeness of the sample; and (4) study results were also analyzed according to smoking status, as a major lifestyle-related source of sCd in the target population.

However, several limitations should be acknowledged: (1) the observational and cross-sectional design precludes any temporal relationships between exposure and outcome, and therefore, missing a potential causal inference; (2) semen samples suitable for sCd measurements by ICP-MS were not available for all participants; however, measurements were performed in 383/493 participants, corresponding to approximately 78% of the total cohort, and all analyses involving sCd were performed within the measured subset, with overtly reported sample sizes for each analysis. Participants with available sCd measurements differed from those without available measurements for some anthropometric and lifestyle-related characteristics; although sCd-related analyses were performed within the measured subset, these differences may limit the generalizability of the findings to the entire recruited cohort; (3) measurement of Cd in whole semen samples prevents distinguishing whether Cd derives from seminal plasma or cells such as spermatozoa, leukocytes, or other cellular components, or precisely inferring the contribution of prostate or seminal vesicle as anatomical sources of Cd within the male reproductive tract [42]. Moreover, objective Cd quantification was performed by ICP-MS only in whole semen samples and not in blood or urine, therefore not allowing us to determine whether higher sCd reflects systemic Cd exposure, exclusive local accumulation in the male reproductive tract, or both [42]; although this may have limited the amount of available information, substantial evidence indicates that Cd selectively accumulates in the testis, and its concentration within the male reproductive tract is not adequately reflected by circulating or urinary concentrations. This suggests that Cd measured in semen is likely the most biologically meaningful indicator of exposure to be considered in reproductive studies [20,24,25]; (4) although the complete-case approach (no imputation) adopted for missing data may have reduced sample sizes, the available sample size is reported for each analysis, and the proportion of missing data is low and similar for subgroups, by reducing the likelihood of biased estimates; (5) although key lifestyle cues were addressed and no significant differences emerged in subgroup comparisons and correlation analyses, the possibility of residual confounding deriving from these factors cannot be entirely excluded, due to the unadjusted analytical approach; (6) residual confounding or modifying effects deriving from exposure to unmeasured complex pollutant mixtures and/or variability in essential trace elements cannot be excluded; and (7) hormonal parameters were not assessed.

## 5. Conclusions

The current single-center, observational, cross-sectional study, nested in a large cohort of males of reproductive age residing in HI areas of the Campania Region—Southern Italy, reported a significant association between sCd levels and impaired semen quality, including significantly reduced sperm total count and normal morphology, with a consensual significantly higher prevalence of pathological seminal parameters and, specifically, of oligozoospermia, in participants with detectable sCd, and, more markedly, in those with sCd level above the median value, compared to participants with undetectable sCd, although subgroup comparisons highlighted a homogeneous profile in major confounders, including age, BMI, smoking habits, duration of residence or mean time spent/day in HI areas, and self-reported occupational exposure.

While these findings do not imply causation, due to the observational and cross-sectional design of the study, results are consistent with and further extend current epidemiological evidence linking Cd exposure to male reproductive dysfunction and are in line with experimental evidence providing biological plausibility for the observed associations. It should be mentioned that no standardized clinical reference interval for sCd is currently available [42]; therefore, the LoD-based and median-based cut-offs used in the current study should be interpreted as analytical or exploratory thresholds. Future studies should adopt longitudinal designs and integrate molecular endpoints to establish temporality and identify the potential causal mechanisms, as well as to define clinical toxicity thresholds, by offering a powerful readout in potential approaches reframing fertility preservation strategies in environmentally exposed male populations.

## Figures and Tables

**Table 1:** Total cohort anthropometric and lifestyle-related parameters.

	Total Cohort (N = 493)
**Age (years)**	29.07 ± 7.17
**BMI (Kg/m^2^)**	25.82 ± 4.1
**Mean residence in HI areas (years)**	25.59 ± 9.13
**Mean time spent in HI areas (h/24 h)**	16.68 ± 5.85
**Non-smokers (n°/%)**	243/52.7 ^a^
**Current smokers (n°/%)**	218/47.3 ^a^
**Cigarettes/day (n°)**	11.02 ± 8.06 ^a^
**Cigarette pack/year (n°)**	6.08 ± 8.47 ^a^
**Occupational exposure to toxic chemicals (n°/%)**	54/11.7 ^a^

Values expressed as mean ± SD or number of cases/percentage. Abbreviations: BMI, body mass index; HI, high environmental impact. ^a^ Determined in 461 participants.

**Table 2:** Total cohort seminal parameters and prevalence of normozoospermia and of pathological seminal parameters.

	Total Cohort (N = 493)
**Semen pH**	8.32 ± 0.3
**Semen Volume (mL)**	3.13 ± 1.67
**Sperm Concentration (n × 10^6^/mL)**	37.58 ± 30.18
**Sperm Total Count (n × 10^6^/ejaculate)**	111.2 ± 104
**Sperm Total Motility (%)**	56.83 ± 16.09
**Sperm Progressive Motility (%)**	50.22 ± 16.63
**Sperm In Situ Motility (%)**	6.72 ± 3.43
**Immotile Spermatozoa (%)**	43.07 ± 15.88
**Sperm Normal Morphology (%)**	7.97 ± 4.02
**Sperm Viability (%)**	64.75 ± 15.34
**Normozoospermia (n°/%)**	328/66.5
**Pathological seminal parameters (n°/%)**	165/33.5
**Oligozoospermia (n°/%)**	69/14
**Cryptozoospermia (n°/%)**	4/0.8
**Azoospermia (n°/%)**	11/2.2
**Asthenozoospermia (n°/%)**	15/3
**Teratozoospermia (n°/%)**	3/0.6
**Oligo-astheno-teratozoospermia (n°/%)**	30/6.1
**Necrozoospermia (n°/%)**	28/5.7
**Different combined seminal parameters alterations (n°/%)**	35/7.1

Values expressed as mean ± SD or number of cases/percentage.

**Table 3:** Anthropometric, lifestyle-related, and seminal parameters, and prevalence of normozoospermia and of pathological seminal parameters in subgroups of participants with detectable and undetectable whole semen cadmium (sCd) level.

	Detectable sCd (N = 128)	Undetectable sCd (N = 255)	*p*
**Age (years)**	30.58 ± 7.5	29.45 ± 7.06	*p* = 0.231
**BMI (Kg/m^2^)**	25.98 ± 3.8	25.99 ± 4.13	*p* = 0.890
**Mean residence in HI areas (years)**	25.32 ± 9.8	26.08 ± 9.18	*p* = 0.468
**Mean time spent in HI areas (h/24 h)**	17.02 ± 5.99	16.52 ± 5.78	*p* = 0.642
**Current smokers (n°/%)**	45/37.8 ^a^	115/47.7 ^b^	*p* = 0.091
**Cigarettes/day (n°)**	12.62 ± 8.34 ^a^	11.17 ± 8.2 ^b^	*p* = 0.285
**Cigarette pack years (n°)**	8.02 ± 8.6 ^a^	6.89 ± 9.61 ^b^	*p* = 0.328
**Occupational exposure to toxic chemicals (n°/%)**	13/10.9 ^a^	36/14.9 ^b^	*p* = 0.331
**Semen pH**	8.38 ± 0.27	8.35 ± 0.24	*p* = 0.239
**Semen volume (mL)**	3.1 ± 1.57	3.34 ± 1.63	*p* = 0.122
**Sperm concentration (n × 10^6^/mL)**	32.44 ± 26.58	35.3 ± 26.29	*p* = 0.231
**Sperm total count (n × 10^6^/ejaculate)**	93.28 ± 84.88	113.2 ± 101.5	***p* = 0.037**
**Sperm total motility (%)**	56.45 ± 16.22	57.86 ± 15.83	*p* = 0.343
**Sperm progressive motility (%)**	49.7 ± 16.97	51.77 ± 16.37	*p* = 0.203
**Sperm in situ motility (%)**	6.75 ± 2.97	6.32 ± 3.05	*p* = 0.129
**Immotile spermatozoa (%)**	43.71 ± 16.17	41.87 ± 15.40	*p* = 0.243
**Sperm normal morphology (%)**	7.29 ± 3.71	8.23 ± 3.91	***p* = 0.034**
**Sperm viability (%)**	68.09 ± 13.02	62.51 ± 14.29	***p* = 0.049**
**Normozoospermia (n°/%)**	77/60.2	184/72.2	***p* = 0.02**
**Pathological seminal parameters (n°/%)**	51/39.8	71/27.8	***p* = 0.02**
**Oligozoospermia (n°/%)**	27/21.1	32/12.6	***p* = 0.036**
**Cryptozoospermia (n°/%)**	1/0.8	2/0.8	*p* > 0.999
**Azoospermia (n°/%)**	0/0	2/0.8	*p* = 0.554
**Asthenozoospermia (n°/%)**	6/4.7	5/2	*p* = 0.192
**Teratozoospermia (n°/%)**	1/0.8	0/0	*p* = 0.334
**Oligo-astheno-teratozoospermia (n°/%)**	10/7.8	13/5.1	*p* = 0.362
**Necrozoospermia (n°/%)**	5/3.9	16/6.3	*p* = 0.471
**Different combined seminal parameters alterations (n°/%)**	8/6.3	18/7.1	*p* = 0.833

Values expressed as mean ± SD or number of cases/percentage. Abbreviations: BMI, body mass index; HI, high environmental impact. ^a^ Determined in 119 participants; ^b^ Determined in 241 participants.

**Table 4:** Anthropometric, lifestyle-related, and seminal parameters, and prevalence of normozoospermia and of pathological seminal parameters in subgroups of participants with whole semen cadmium (sCd) level above the median value, sCd level below the median value, and undetectable sCd level.

	sCd Above Median Value (N = 98)	sCd Below Median Value (N = 30)	Undetectable sCd (N = 255)	*p*
**Age (years)**	30.61 ± 7.7	30.47 ± 6.93	29.45 ± 7.06	*p* = 0.488
**BMI (Kg/m^2^)**	26 ± 4.08	25.9 ± 2.78	25.99 ± 4.13	*p* = 0.971
**Mean residence in HI areas (years)**	26.20 ± 9.1	22.43 ± 11.78	26.08 ± 9.18	*p* = 0.314
**Mean time spent in HI areas (h/24 h)**	17.19 ± 5.75	16.47 ± 6.81	16.52 ± 5.78	*p* = 0.698
**Current smokers (n°/%)**	35/37.6 ^a^	10/38.5 ^b^	115/47.7 ^c^	*p* = 0.205
**Cigarettes/day (n°)**	12.74 ± 8.14 ^a^	12.2 ± 9.46 ^b^	11.17 ± 8.2 ^c^	*p* = 0.533
**Cigarette pack years (n°)**	7.6 ± 7.62 ^a^	9.51 ± 11.78 ^b^	6.89 ± 9.61 ^c^	*p* = 0.586
**Occupational exposure to toxic chemicals (n°/%)**	11/11.8 ^a^	2/7.7 ^b^	36/14.9 ^c^	*p* = 0.5
**Semen pH**	8.38 ± 0.28	8.38 ± 0.23	8.35 ± 0.24	*p* = 0.499
**Semen volume (mL)**	3.19 ± 1.58	2.81 ± 1.55	3.34 ± 1.63	*p* = 0.141
**Sperm concentration (n × 10^6^/mL)**	29.12 ± 24.84 ^1,2^	43.62 ± 29.55 ^1^	35.3 ± 26.29 ^2^	***p* = 0.017**
**Sperm total count (n × 10^6^/ejaculate)**	85.77 ± 80.52 ^3^	117.8 ± 95.16	113.2 ± 101.5 ^3^	***p* = 0.024**
**Sperm total motility (%)**	56.51 ± 15.5	56.24 ± 18.73	57.86 ± 15.83	*p* = 0.638
**Sperm progressive motility (%)**	49.77 ± 16.21	49.48 ± 19.64	51.77 ± 16.37	*p* = 0.445
**Sperm in situ motility (%)**	6.75 ± 2.92	6.76 ± 3.18	6.32 ± 3.05	*p* = 0.310
**Immotile spermatozoa (%)**	43.59 ± 15.43	44.1 ± 18.73	41.87 ± 15.40	*p* = 0.502
**Sperm normal morphology (%)**	6.92 ± 3.38 ^4^	8.55 ± 4.49	8.23 ± 3.91 ^4^	***p* = 0.015**
**Sperm viability (%)**	68.17 ± 12.65	67.89 ± 14.75	62.51 ± 14.29	*p* = 0.130
**Normozoospermia (n°/%)**	56/57.1 *	21/70	184/72.2 *	***p* = 0.025**
**Pathological seminal parameters (n°/%)**	42/42.9 *	9/30	71/27.8 *	***p* = 0.025**
**Oligozoospermia (n°/%)**	23/23.5 ^§^	4/13.3	32/12.6 ^§^	***p* = 0.037**
**Cryptozoospermia (n°/%)**	0/0	1/3.3	2/0.8	*p* = 0.194
**Azoospermia (n°/%)**	0/0	0/0	2/0.8	*p* = 0.604
**Asthenozoospermia (n°/%)**	5/5.1	1/3.3	5/1.9	*p* = 0.282
**Teratozoospermia (n°/%)**	1/1.02	0/0	0/0	*p* = 0.233
**Oligo-astheno-teratozoospermia (n°/%)**	7/7.1	3/10	13/5.1	*p* = 0.486
**Necrozoospermia (n°/%)**	4/4.1	1/3.3	16/6.3	*p* = 0.623
**Different combined seminal parameters alterations (n°/%)**	8/8.2	0/0	18/7.1	*p* = 0.286

Values expressed as mean ± SD or number of cases/percentage. Abbreviations: BMI, body mass index; HI, high environmental impact. ^a^ Determined in 93 participants; ^b^ Determined in 26 participants; ^c^ Determined in 241 participants. ^1^ Significantly different, *p* = 0.015; ^2^ Significantly different, *p* = 0.03; ^3^ Significantly different, *p* = 0.008; ^4^ Significantly different, *p* = 0.006; * Significantly different, *p* = 0.008; ^§^ Significantly different, *p* = 0.014.

**Table 5:** Anthropometric, lifestyle-related, and seminal parameters, and prevalence of normozoospermia and of pathological seminal parameters in subgroups of smoker and non-smoker participants with detectable whole semen cadmium (sCd) level.

	Smokers (N = 45)	Non-Smokers (N = 74)	*p*
**Age (years)**	30.36 ± 7.98	30.41 ± 7.28	*p* = 0.807
**BMI (Kg/m^2^)**	25.35 ± 3.07	26.48 ± 4.28	*p* = 0.272
**Mean residence in HI areas (years)**	24.8 ± 9.66	26.12 ± 9.52	*p* = 0.530
**Mean time spent in HI areas (h/24 h)**	16.02 ± 5.48	17.24 ± 6.22	*p* = 0.250
**Occupational exposure to toxic chemicals (n°/%)**	7/15.6	6/8.1	*p* > 0.999
**Semen pH**	8.49 ± 0.24	8.33 ± 0.27	*p* = 0.3
**Semen volume (mL)**	2.98 ± 1.53	3.3 ± 1.64	*p* = 0.141
**Sperm concentration (n × 10^6^/mL)**	27.97 ± 21.54	34.78 ± 28.54	*p* = 0.347
**Sperm total count (n × 10^6^/ejaculate)**	81.33 ± 80.83	103.3 ± 88.59	*p* = 0.209
**Sperm total motility (%)**	55.02 ± 15.1	58.15 ± 16.37	*p* = 0.214
**Sperm progressive motility (%)**	48.09 ± 16.09	51.54 ± 16.97	*p* = 0.2
**Sperm in situ motility (%)**	6.93 ± 2.88	6.61 ± 3.15	*p* = 0.403
**Immotile spermatozoa (%)**	44.98 ± 15.08	42.12 ± 16.31	*p* = 0.257
**Sperm normal morphology (%)**	6.66 ± 3.54	7.73 ± 3.8	*p* = 0.058
**Sperm viability (%)**	72.86 ± 10.38	62.4 ± 14.19	*p* = 0.063
**Normozoospermia (n°/%)**	24/53.3	49/66.2	*p* = 0.179
**Pathological seminal parameters (n°/%)**	21/46.7	25/33.8	*p* = 0.179
**Oligozoospermia (n°/%)**	12/26.7	14/18.1	*p* = 0.364
**Cryptozoospermia (n°/%)**	1/2.2	0/0	*p* = 0.378
**Azoospermia (n°/%)**	0/0	0/0	*p* > 0.999
**Asthenozoospermia (n°/%)**	2/4.4	3/4.1	*p* > 0.999
**Teratozoospermia (n°/%)**	0/0	1/1.4	*p* > 0.999
**Oligo-astheno-teratozoospermia (n°/%)**	2/4.4	6/8.2	*p* = 0.709
**Necrozoospermia (n°/%)**	0/0	5/6.8	*p* = 0.156
**Different combined seminal parameters alterations (n°/%)**	4/8.8	3/4.1	*p* = 0.424

Values expressed as mean ± SD or number of cases/percentage. Abbreviations: BMI, body mass index; HI, high environmental impact.

## Data Availability

The datasets generated during and/or analyzed during the current study are available from the corresponding author on reasonable request.

## Supplementary Materials

The following supporting information can be downloaded at: https://www.mdpi.com/article/10.3390/jcm15134949/s1. Table S1. Anthropometric, lifestyle-related and seminal parameters, in subgroups of participants with unavailable and available whole semen cadmium (sCd) measurement. Values expressed as mean ± SD or percentage. Abbreviations: BMI, body mass index. Table S2. Values are reported as mean ± SD or n/N (%). Mean differences and risk differences were calculated as detectable sCd minus undetectable sCd. Risk differences are expressed as percentage points. For risk differences, 95% confidence intervals were calculated using the Newcombe-Wilson method. Table S3. Values are reported as mean ± SD or n/N (%). Mean differences and risk differences were calculated using undetectable sCd as the reference category. Risk differences are expressed as percentage points. For risk differences, 95% confidence intervals were calculated using the Newcombe-Wilson method.
