# Supplementary material for: Semen Quality in a Large Cohort of Males Living in Highly Polluted Areas of Campania Region in Southern Italy with a Focus on the Role of Cadmium Exposure
Source: J Clin Med. 2026 Jun 25;15(13):4949. doi: 10.3390/jcm15134949 (PMC13361313; doi:10.3390/jcm15134949)
Supplement: Supplementary file 1 [file jcm-15-04949-s001.zip › jcm-4265269-supplementary.pdf]

	Unavailable sCd measurement (N=110)	Available sCd measurement (N=383)	p
**Age (years)**	26.72 ± 6.65	29.83 ± 7.21	***p<0.001***
**BMI (Kg/m²)**	25.29 ± 4.36	25.98 ± 4.01	***p=0.047***
**Pack years**	2.6 ± 3.36	7.14 ± 9.32	***p<0.001***
**Occupational exposure to toxic chemicals (%)**	5.5	14.1	***p=0.03***
**Sperm Total Count (nx10⁶/ejaculate)**	128.8 ± 127.4	106.5 ± 95.58	p=0.302
**Sperm Total Motility (%)**	54.7 ± 16.52	57.39 ± 15.95	p=0.129
**Sperm Progressive Motility (%)**	47.02 ± 16.51	51.08 ± 16.58	***p=0.026***
**Sperm In Situ Motility (%)**	7.68 ± 4.51	6.46 ± 3.03	p=0.055
**Immotile Spermatozoa (%)**	42.25 ± 16.54	42.49 ± 15.66	p=0.135
**Sperm Normal Morphology (%)**	8.18 ± 4.57	7.92 ± 3.87	p=0.910
**Sperm Viability (%)**	65.32 ± 20.17	64.6 ± 14.02	p=0.603

Supplementary Table S1. Anthropometric, lifestyle-related and seminal parameters, in subgroups of participants with unavailable and available whole semen cadmium (sCd) measurement. Values expressed as mean ± SD or percentage. Abbreviations: BMI, body mass index.

	Detectable sCd	Undetectable sCd	Mean/Risk difference (95% CI)
**Semen pH**	8.38 ± 0.27	8.35 ± 0.24	0.03 (-0.03 to 0.08)
**Semen Volume (ml)**	3.10 ± 1.57	3.34 ± 1.63	-0.25 (-0.59 to 0.09)
**Sperm Concentration (nx10^6/ml)**	32.44 ± 26.58	35.30 ± 26.29	-2.86 (-8.53 to 2.81)
**Sperm Total Count (nx10^6/ejaculate)**	93.28 ± 84.88	113.16 ± 101.49	-19.88 (-39.26 to -0.49)
**Sperm Total Motility (%)**	56.45 ± 16.22	57.86 ± 15.83	-1.41 (-4.86 to 2.03)
**Sperm Progressive Motility (%)**	49.70 ± 16.97	51.77 ± 16.37	-2.07 (-5.66 to 1.53)
**Sperm In Situ Motility (%)**	6.75 ± 2.97	6.32 ± 3.05	0.43 (-0.21 to 1.07)
**Immotile Spermatozoa (%)**	43.71 ± 16.17	41.87 ± 15.40	1.84 (-1.58 to 5.25)
**Sperm Normal Morphology (%)**	7.29 ± 3.71	8.23 ± 3.91	-0.94 (-1.75 to -0.13)
**Sperm Viability (%)**	68.09 ± 13.02	62.51 ± 14.29	5.58 (-0.35 to 11.51)
**Normozoospermia (%)**	77/128 (60.2)	184/255 (72.2)	-12.00 (-22.07 to -2.07)
**Seminal parameters below reference cut-off values (any) (%)**	51/128 (39.8)	71/255 (27.8)	12.00 (2.07 to 22.07)
**Oligozoospermia (%)**	27/128 (21.1)	32/255 (12.5)	8.54 (0.83 to 17.15)
**Cryptozoospermia (%)**	1/128 (0.8)	2/255 (0.8)	-0.00 (-2.13 to 3.55)
**Azoospermia (%)**	0/128 (0.0)	2/255 (0.8)	-0.78 (-2.81 to 2.18)
**Asthenozoospermia (%)**	6/128 (4.7)	5/255 (2.0)	2.73 (-0.86 to 8.01)
**Teratozoospermia (%)**	1/128 (0.8)	0/255 (0.0)	0.78 (-0.84 to 4.29)
**Oligo-astheno-teratozoospermia (%)**	10/128 (7.8)	13/255 (5.1)	2.71 (-2.19 to 9.04)
**Necrozoospermia (%)**	5/128 (3.9)	16/255 (6.3)	-2.37 (-6.66 to 3.09)
**Other combined seminal parameters alterations (%)**	8/128 (6.3)	18/255 (7.1)	-0.81 (-5.70 to 5.34)

Supplementary Table S2. Values are reported as mean ± SD or n/N (%). Mean differences and risk differences were calculated as detectable sCd minus undetectable sCd. Risk differences are expressed as percentage points. For risk differences, 95% confidence intervals were calculated using the Newcombe-Wilson method.

	sCd above median value	sCd below median value	Undetectable sCd	Difference: above median vs undetectable (95% CI)	Mean/Risk difference: below median vs undetectable (95% CI)
**Semen pH**	8.38 ± 0.28	8.38 ± 0.23	8.35 ± 0.24	0.03 (-0.03 to 0.09)	0.03 (-0.06 to 0.12)
**Semen Volume (ml)**	3.19 ± 1.58	2.81 ± 1.55	3.34 ± 1.63	-0.16 (-0.53 to 0.22)	-0.53 (-1.14 to 0.08)
**Sperm Concentration (nx10^6/ml)**	29.12 ± 24.84	43.62 ± 29.55	35.30 ± 26.29	-6.17 (-12.10 to -0.25)	8.33 (-3.33 to 19.98)
**Sperm Total Count (nx10^6/ejaculate)**	85.77 ± 80.52	117.81 ± 95.16	113.16 ± 101.49	-27.39 (-47.76 to -7.01)	4.65 (-32.84 to 42.14)
**Sperm Total Motility (%)**	56.51 ± 15.50	56.24 ± 18.73	57.86 ± 15.83	-1.35 (-5.01 to 2.31)	-1.62 (-8.98 to 5.74)
**Sperm Progressive Motility (%)**	49.77 ± 16.21	49.48 ± 19.64	51.77 ± 16.37	-2.00 (-5.82 to 1.81)	-2.29 (-10.00 to 5.43)
**Sperm In Situ Motility (%)**	6.74 ± 2.92	6.76 ± 3.18	6.32 ± 3.05	0.43 (-0.27 to 1.12)	0.44 (-0.82 to 1.70)
**Immotile Spermatozoa (%)**	43.59 ± 15.43	44.10 ± 18.73	41.87 ± 15.40	1.72 (-1.90 to 5.34)	2.23 (-5.12 to 9.58)
**Sperm Normal Morphology (%)**	6.92 ± 3.38	8.55 ± 4.49	8.23 ± 3.91	-1.31 (-2.14 to -0.48)	0.32 (-1.45 to 2.09)
**Sperm Viability (%)**	68.17 ± 12.65	67.89 ± 14.75	62.51 ± 14.29	5.66 (-0.82 to 12.13)	5.38 (-6.30 to 17.06)
**Normozoospermia (%)**	56/98 (57.1)	21/30 (70.0)	184/255 (72.2)	-15.01 (-26.16 to -4.02)	-2.16 (-20.76 to 12.38)
**Seminal parameters below reference cut-off values (any) (%)**	42/98 (42.9)	9/30 (30.0)	71/255 (27.8)	15.01 (4.02 to 26.16)	2.16 (-12.38 to 20.76)
**Oligozoospermia (%)**	23/98 (23.5)	4/30 (13.3)	32/255 (12.5)	10.92 (2.28 to 20.86)	0.78 (-8.48 to 17.51)
**Cryptozoospermia (%)**	0/98 (0.0)	1/30 (3.3)	2/255 (0.8)	-0.78 (-2.81 to 3.03)	2.55 (-0.86 to 15.90)
**Azoospermia (%)**	0/98 (0.0)	0/30 (0.0)	2/255 (0.8)	-0.78 (-2.81 to 3.03)	-0.78 (-2.81 to 10.58)
**Asthenozoospermia (%)**	5/98 (5.1)	1/30 (3.3)	5/255 (2.0)	3.14 (-0.72 to 9.53)	1.37 (-2.37 to 14.76)
**Teratozoospermia (%)**	1/98 (1.0)	0/30 (0.0)	0/255 (0.0)	1.02 (-0.68 to 5.56)	0.00 (-1.48 to 11.35)
**Oligo-astheno-teratozoospermia (%)**	7/98 (7.1)	3/30 (10.0)	13/255 (5.1)	2.04 (-2.96 to 9.23)	4.90 (-2.48 to 20.66)
**Necrozoospermia (%)**	4/98 (4.1)	1/30 (3.3)	16/255 (6.3)	-2.19 (-6.63 to 4.21)	-2.94 (-7.53 to 10.61)

**Other combined seminal parameters alterations (%)**	8/98 (8.2)	0/30 (0.0)	18/255 (7.1)	1.10 (-4.41 to 8.67)	-7.06 (-10.88 to 4.57)

Supplementary Table S3. Values are reported as mean ± SD or n/N (%). Mean differences and risk differences were calculated using undetectable sCd as the reference category. Risk differences are expressed as percentage points. For risk differences, 95% confidence intervals were calculated using the Newcombe-Wilson method.
